# Dietary Propolis Ameliorates Dextran Sulfate Sodium-Induced Colitis and Modulates the Gut Microbiota in Rats Fed a Western Diet

**DOI:** 10.3390/nu9080875

**Published:** 2017-08-14

**Authors:** Kai Wang, Xiaolu Jin, Mengmeng You, Wenli Tian, Richard K. Le Leu, David L. Topping, Michael A. Conlon, Liming Wu, Fuliang Hu

**Affiliations:** 1 Institute of Apiculture Research, Chinese Academy of Agricultural Sciences, Beijing 100093, China; kaiwang628@gmail.com (K.W.); leroytian@126.com (W.T.); 2 College of Animal Sciences, Zhejiang University, Hangzhou 310058, China; ymm0233@163.com; 3 CSIRO Health and Biosecurity, Adelaide, SA 5000, Australia; david.topping@csiro.au; 4 Beijing Advanced Innovation Center for Food Nutrition and Human Health, College of Food Science and Nutritional Engineering, China Agricultural University, Beijing 100083, China; chnfhjxl@163.com; 5 Central and Northern Adelaide Renal and Transplantation Service, Royal Adelaide Hospital, Adelaide, SA 5000, Australia; richard.leleu@sa.gov.au

**Keywords:** propolis, rats, colitis, gut microbiota

## Abstract

Propolis is an important hive product and considered beneficial to health. However, evidence of its potential for improving gut health is still lacking. Here we use rats to examine whether dietary supplementation with propolis could be used as a therapy for ulcerative colitis. Rats were fed with a Western style diet alone (controls) or supplemented with different amounts of Chinese propolis (0.1%, 0.2%, and 0.3%) to examine effects on acute colitis induced by 3% dextran sulphate sodium (DSS) in drinking water. Propolis at 0.3%, but not lower levels, significantly improved colitis symptoms compared with the control group, with a less pronounced disease activity index (DAI) (*p* < 0.001), a significant increase in colon length/weight ratio (*p* < 0.05) and an improved distal colon tissue structure as assessed by histology. Although short chain fatty acid levels in digesta were not altered by propolis supplementation, 16S rRNA phylogenetic sequencing revealed a significant increase in gut microbial diversity after 21 days of 0.3% propolis supplementation compared with controls including a significant increase in bacteria belonging to the Proteobacteria and Acidobacteria phyla. This is the first study to demonstrate that propolis can attenuate DSS-induced colitis and provides new insight into diet-microbiota interactions during inflammatory bowel disease.

## 1. Introduction

Inflammatory bowel disease (IBD), which includes Crohn’s disease (CD) and ulcerative colitis (UC), is a chronic disorder of the gastrointestinal tract characterized by inflammation. Individuals with UC typically suffer with recurrent inflammation of the colon whereas in CD patients the inflammatory responses can occur in the terminal ileum and colon [1]. Although the precise aetiology of IBD still remains unknown, and there are some cases reporting genetic susceptibility of some individuals, a dysbiosis of the gut microbiota remains a consistent underlying feature [2]. Environmental factors, diet and lifestyle have also been widely recognized as potential triggers for IBD. Recent rapid increases in the incidence of IBD in developing countries have occurred along with considerable shifts toward Western style dietary habits [3]. Dietary intervention is also being considered as a means of IBD prevention or treatment, due to the significant influence it has on the metabolism and diversity of gut microbiota populations [4,5].

Recent studies suggest there are beneficial effects of polyphenol-rich foods on human health, and may provide promising alternative approaches to preventing or treating chronic diseases, including IBD [6]. Polyphenolic constituents which possess potent antioxidant activities function not only as free-radical scavengers, but also modulators of cellular redox signalling pathways during different physiological/pathological stages. Their anti-inflammatory activities are also widely acknowledged since they inhibit the production and the release of several inflammatory mediators like interleukins, tumour necrosis factor, nitric oxide, etc. [6]. More importantly, recent evidence suggests that dietary polyphenols modulate human intestinal microbial populations due to their bacteriostatic or bactericidal actions [7], which could be of particular value for treating IBD given the apparent role of gut microbes as a contributor to the condition.

Propolis is a resinous substance collected by honeybees, *Apis mellifera*, from various plant sources, which contains abundant polyphenolic compounds [8]. Our previous studies using different animal models showed that polyphenol-rich propolis extracts exhibited significant anti-inflammatory effects, probably via modulating the production of key inflammatory mediators and by blocking the activation of NF-κB [9,10]. Propolis’ chemical properties vary slightly depending on the geographic location from which it is derived, but a broad spectrum of bioactivities including anti-inflammatory effects are consistently present, suggesting it could be used to treat diseases, such as IBD. 

Here, we describe a study to determine whether the addition of a Chinese propolis extract to a Western style diet can reduce the severity of colitis induced by dextran sulphate sodium (DSS) in rats and using this model to examine the contribution that the gut microbiota may play in this protection. The major shifts in population of microbiota in DSS-induced colitis in rats are similar to those in human UC [11,12] and we will examine the contribution that the gut microbiota may play in any protection observed with dietary propolis in this model.

## 2. Materials and Methods

### 2.1. Animals and Treatments

Male Sprague Dawley rats (six-week old, ~185 g) were obtained from Laboratory Animal Services, University of Adelaide, Australia. Animal experimental procedures were approved by the CSIRO Animal Ethics Committee (SA), Australia and carried out in accordance with the code for the care and use of animals for scientific purposes (ethic approval code: 803-12/15). Throughout the study they were housed in stainless-steel cages with wire-mesh bottoms within a temperature (22 ± 2 °C) and humidity-controlled (80 ± 10%) animal facility with a 12 h light/dark cycle and had free access to feed and water. Rats were acclimatized for seven days before the experimental diets started. During acclimatization rats consumed a standard commercially-available chow. The animals were randomly divided into four treatment groups of equal size (*n* = 8/group) with comparable initial body weights. The experimental diets were a modification of the AIN 93G diet in which protein and fat levels were increased to 25% and 20%, respectively, to approximate a Western style diet (Table 1) which was supplemented with 0% (control), 0.1%, 0.2%, or 0.3% of a Chinese propolis extract. The Chinese propolis extract (poplar type) was obtained from the same batch used in a previously described study and for which the chemical constituents were analysed [13]. Propolis was added to the diet by first dissolving it in the oil used as the fat source. The diets were fed in a pelleted form. Rats consumed the experimental diets for 21 days. After the first seven days on the experimental diets, dextran sulphate sodium (DSS; molecular weight 36–50 kDa; MP Biomedicals) was added to the drinking water at a level of 3% for a period of seven days, after which all rats again received standard tap water without DSS for another seven days. At the completion of the 21 days of the experimental diets rats were anaesthetised with 4% isofluorane/oxygen and killed to enable tissue collection. An additional five rats which did not receive DSS but which did receive the Western style diet containing the control (0% DSS) diet were also included in the study and killed along with the other rats.

A limited repeat of the above study (carried out in Australia) was performed in China using the same methods for the purpose of obtaining digesta for microbiota and histology analyses. The China-based activities were approved by the Animal Care and Use Committee of Zhejiang University, China, and the animals provided by the Laboratory Animal Services Centre, Zhejiang University (ethic approval code: AEC-160301). In that study twenty-four rats were randomly divided into three equal groups (*n* = 8/group). One group received 21 days of 0% propolis supplementation of the Western style diet and another 0.3% propolis supplementation. The remaining group received the Western style diet with 0% propolis but did not receive any DSS treatment.

### 2.2. Disease Activity Index (DAI) Observations

A disease activity index (DAI) based on bodyweight change, stool consistency, rectal bleeding and overall condition of the animal [14] was calculated daily from the start of DSS treatment to the end of the experiment. Scores were recorded using following criteria: (a) weight loss: 0—no weight loss, 1—a weight loss of between 0.1% and 5%, 2—a weight loss of between 5% and 10%, 3—a weight loss of more than 10%; (b) Stool consistency: 0—normal, 1—loose stool (regular shape, wet), 2—mild diarrhoea (less regular shape, pasty), 3—diarrhoea (no shape, very wet); (c) rectal bleeding: 0—No observable blood, 1—small amount of blood in some stool, 2—blood in stool regularly seen, 3—blood in all stool; and (d) overall condition of the animal (including vitality, coat condition, posture, behaviour): 0—normal, 1—some signs of poor condition, 2—moderately poor condition, 3—very poor condition.

### 2.3. Histological Analyses

For histological examination, distal colon tissues were flushed with chilled phosphate-buffered saline (PBS, pH 7.2) fixed in neutral buffered formalin, dehydrated in a graded ethanol series and embedded in paraffin. Tissue sections were stained with haematoxylin and eosin (H and E) and observed under a light microscopy. Colonic histological damage was scored based on two subscores (cell infiltration and tissue damage), ranging from 0–6 (no changes to extensive cell infiltration and tissue damage) [15]. Infiltration of inflammatory cells were counted as 0 (rare inflammatory cells in the lamina propria) to 3 (transmural extension of the infiltration of inflammatory cells). Tissue damage were counted as 0 (no mucosal damage) to 3 (extensive mucosal damage and extension through deeper structures of the bowel wall).

### 2.4. Short Chain Fatty Acids (SCFA) Analysis

Weighed portions of cecal digesta collected from rats at the time of kill were diluted at 1:3 (*w/w*) with deionised water containing 1.68 mmol heptanoic acid/L as an internal standard (Sigma Chemical Co., St. Louis, MO, USA). Acetate, butyrate, propionate, and the total SCFA (including minor SCFA) were determined in the caecal contents of rats as described previously using a gas chromatography system as reported previously [16].

### 2.5. Measurement of the Gut Microbiota

DNA was extracted from the caecal digesta of rats in the limited animal study performed in China using the QIAamp DNA stool MiniKit (Qiagen, Hilden, Germany). Bacterial 16S rRNA gene sequences (V3–V4 region) were amplified using Premix Ex TaqTM Hot Start Version (Takara, Dalian, China), and using the following universal primers: 319F (5′-ACTCCTACGGGAGGCAGCAG-3′) and 806R (5′-GGACTACHVGGGTWTCTAAT-3′). Each Polymerase chain reaction (PCR) mixture was prepared in a final 50 μL volume containing 12.5 μL of the Master Mix, 1 μM of each primer, 50 ng of template DNA, and PCR-grade water. PCR reactions were carried out using a gradient PCR instrument (L96G; LongGene, Hangzhou, China). MiSeq Illumina sequencing was further processed on the sequencing reaction (Illumina Inc., San Diego, CA, USA) for paired-end reads. Then the paired-end reads were assembled were merged using FLASH, then assigned to each sample according to the unique barcodes. High-quality tags were clustered into operational taxonomic units (OTUs) using Usearch in QIIME software based on a 97% sequence similarity, and these OTUs were further subjected to analysis using database of Greengenes by RDP algorithm. Alpha and beta diversity and principal coordinate analysis (PCoA) analysis were analysed using QIIME. Linear discriminant analysis (LDA) effect size (LEfSe) analyses were performed with the LEfSe tool [17].

### 2.6. Statistical Analyses

Data on bodyweights, organ weights, DAI, and digesta SCFA are presented as the arithmetic mean ± SEM for each treatment group. The effect of treatments was determined by one-way ANOVA and differences between treatments were analysed post-hoc by Tukey’s honest significant difference test. *p*-values of less than 0.05 were considered statistically significant. All statistical tests were carried using SPSS version 17.0.

## 3. Results

### 3.1. Effects of Propolis on Colitis Symptoms

**Figure 1:**
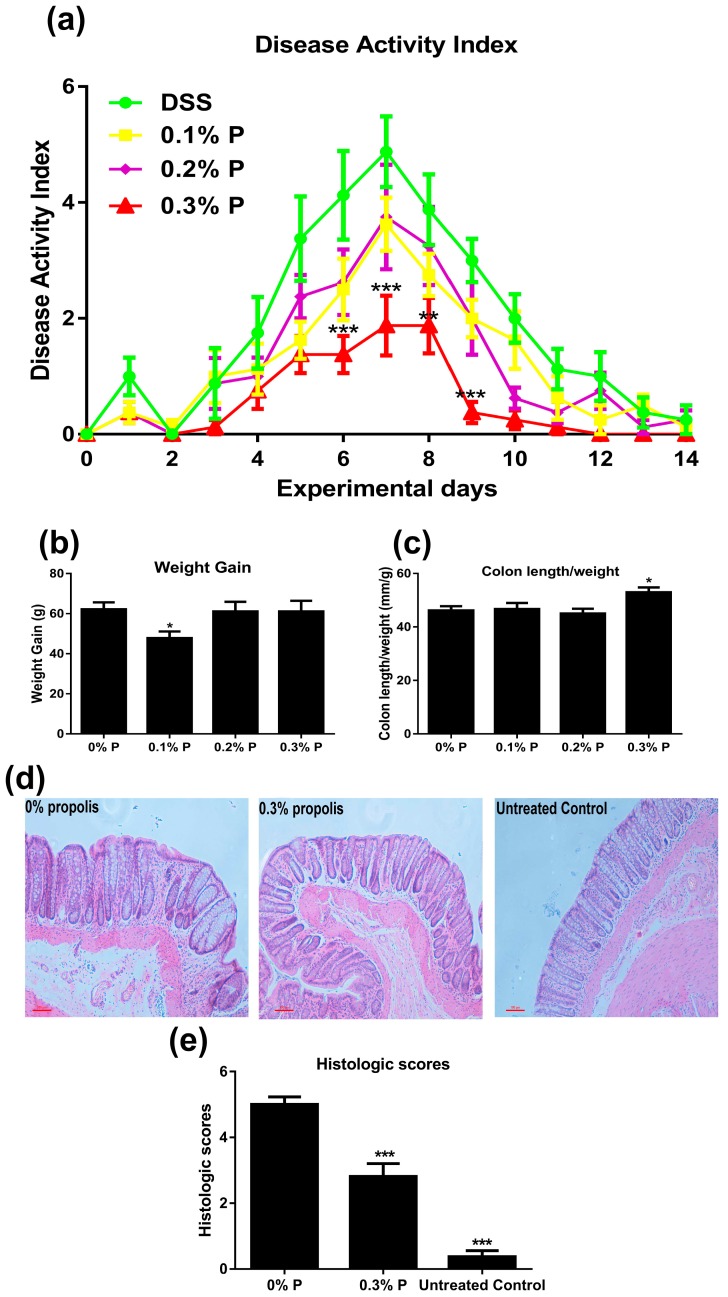
Effects of propolis administration on severity of DSS-induced colitis. Rats were fed with Western diet containing different levels of propolis (0%, 0.1%, 0.2%, and 0.3%) starting for one week before experiment treatment (DSS, MW 36–50 kDa, 3% in the drinking water) started. (**a**) Disease activity index (DAI) over the period of experiment; (**b**) weight gain at the end of the experiment; (**c**) colon length/weight ratio was calculated at the end of the experiment; (**d**) Representative H and E-stained sections from 0% propolis, 0.3% propolis, and the untreated control group; (**e**) The histologic scores of rats (0 for no inflammation, 6 for maximal tissue damage and cell infiltration). The data represent the mean ± SEM of eight rats per group. * *p* < 0.05, and *** *p* < 0.001 significant difference versus the 0% propolis group.

The daily DAI of rats in each of the treatment groups is shown in Figure 1a. DSS treatment significantly increased DAI (*p* < 0.001). We also measured the DAI of untreated controls but the values were zero (data now shown). Administration of 0.3% propolis significantly lowered the DAI (*p* < 0.01 or *p* < 0.001) (D6 to D9). Rats fed 0.1% propolis had significantly lower weight gain than controls during DSS treatment but the 0.2% and 0.3% doses of propolis did not alter weight gain in rats during this period. No significant differences in final bodyweights were observed (Figure 1b) nor did any treatment alter the weights (as a proportion of body weight) of the liver, spleen, kidneys and fat pads (Table 2). However, the 0.3% propolis-fed rats had a significantly greater colon length/weight ratio than untreated controls (Figure 1c, *p* < 0.05). For reference, the body weight gain data, colon length/weight ratio and organ weight from untreated control rats are shown in the Supplementary Table S1. Histological examination of the distal colon of rats treated with DSS showed severe microscopic inflammation of the mucosa, as indicated by ulceration, oedema, crypt damage, and infiltration the intestinal epithelial layer. Compared to rats in the DSS (0% propolis) group, 0.3% propolis reduced histologic evidence of DSS-induced colitis (Figure 1d). 

### 3.2. Effects on SCFA Production

Caecal digesta SCFA levels are shown in Table 3. Addition of propolis to the diet resulted in significantly lower caecal pools of acetate and total SCFA in rats consuming 0.2% propolis when compared to the 0.1% propolis group. No other differences in SCFA levels were observed. Caecal tissue weight, digesta weight and pH were unaffected by propolis treatment. For reference, the caecal digesta SCFA, tissue weight, digesta weight, and pH from untreated control rats are shown in the Supplementary Table S2.

### 3.3. Propolis Altered the Composition of the Intestinal Microbiota in Colitis Rats 

**Figure 2:**
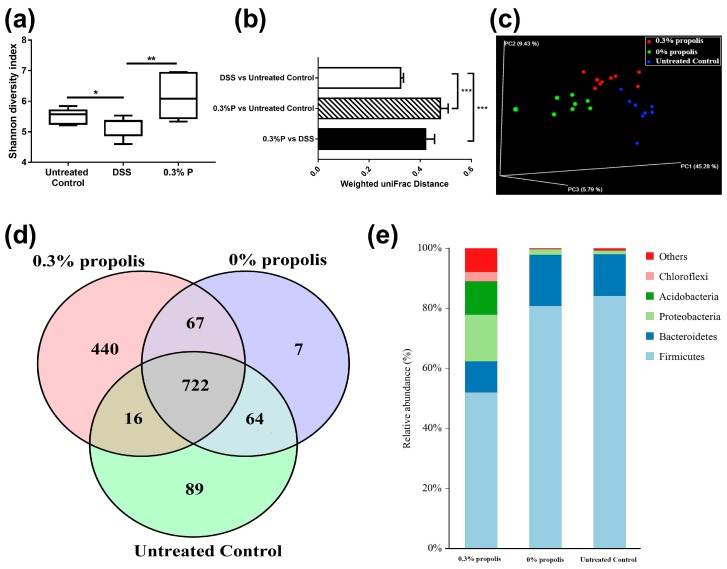
Propolis altered the composition of the intestinal microbiota in colitis rats. Rats’ caecal digesta from untreated control, 0% propolis and 0.3% propolis groups were collected and the intestinal microbiota were examined by 16S rRNA sequencing. (**a**) Alpha diversity was analysed by Shannon diversity index; Data are presented as box plots, *n* = 8 rats/group; * *p* < 0.05, and ** *p* < 0.01 significant difference among two groups; (**b**) Beta diversity between the groups was analysed by weighted UniFrac distance. Data are the mean ± SEM (*n* = 8). *** *p* < 0.001 significant difference among two groups; (**c**) PCoA plot of the gut microbiota based on an unweighted UniFrac metric; (**d**) Venn diagram of OTUs showed microbiota differences between untreated control (green area) and 0% propolis (red area) and between 0.3% propolis (blue area), showing strains altered in these groups, and the overlap among them; and (**e**) relative abundance of predominant bacteria at the phylum level. Data are presented as a staked bar chart, *n* = 8 per group.

16S rRNA phylogenetic approach was used to compare caecal microbial populations of the 0% propolis, 0.3% propolis and non-DSS treated rats. A mean of 37,362 (20,793–51,692) effective tags were observed following sequencing. Sequences with at least 97% similarity were clustered into Operational Taxonomic Units (OTUs). The alpha diversity of microbial communities, as indicated by the Shannon index, was reduced significantly by DSS treatment and propolis significantly increased the microbial diversity (*p* < 0.05, Figure 2a). We also assessed the microbial communities based on the weighted UniFrac distances among the untreated control, 0% propolis and 0.3% propolis groups, obtained data clearly revealed showed propolis supplement caused a significant difference in microbial beta diversity (*p* < 0.001, Figure 2b). Additionally, the structure of the gut microbiota was further studied by weighted PCoA analysis and we noticed that gut microbiota was clearly modulated by propolis supplement and DSS (Figure 2c). A Venn diagram (Figure 2d), displaying the overlapping OTU data for the three groups, shows that 722 of 1405 OTUs accounting for the total richness were universal to all the samples, and that 440 OTUs of the 0.3% propolis group were distinct from the other two groups. At a phylum level, *Firmicutes* and *Bacteroidetes* were predominant in the DSS (0% propolis) and untreated control groups, with only a very small proportion of other phyla. However, these two phyla represented a much smaller proportion of the microbes in the 0.3% propolis treatment group (Figure 2e) with Proteobacteria, Acidobacteria and other microbes increasing to constitute a greater relative proportion of the population. 

### 3.4. Key Phylotypes of Gut Microbiota Altered by Propolis Supplement in Colitis Rats

**Figure 3:**
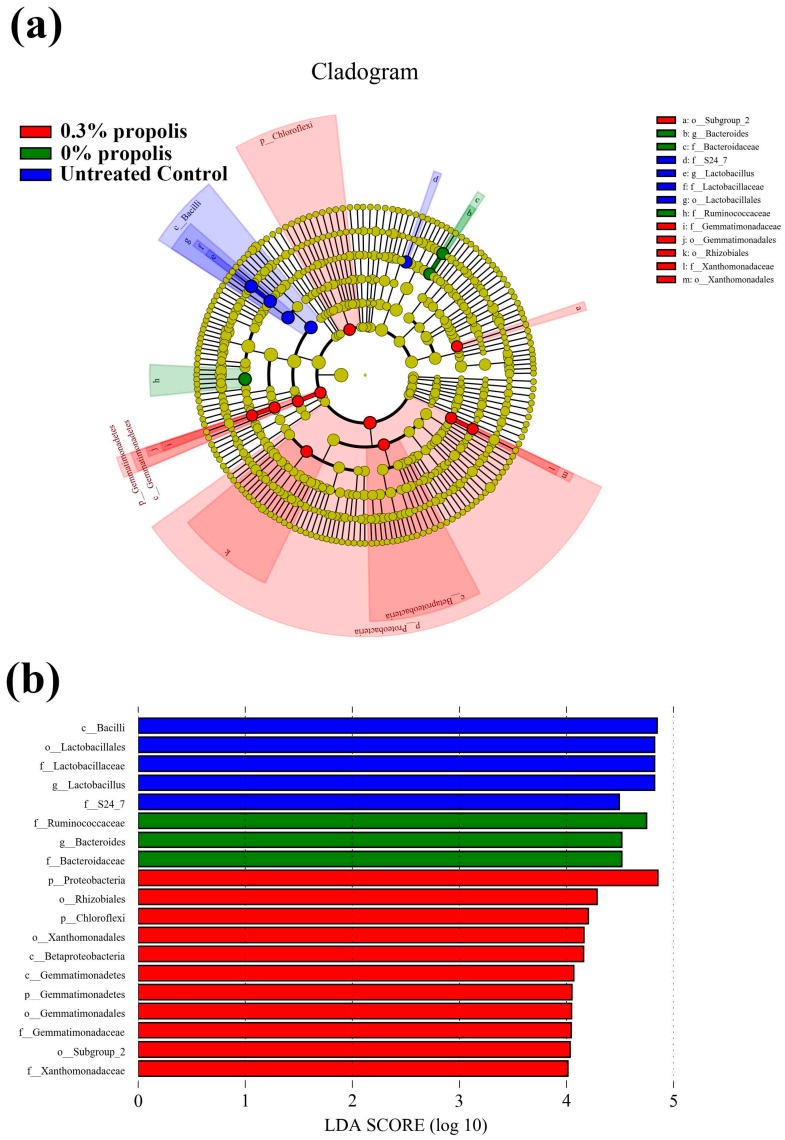
Characteristics of microbial community composition using LEfSe analysis. (**a**) Taxonomic cladogram obtained from LEfSe analysis of 16S rRNA sequencing. Red shaded areas indicate the 0.3% propolis supplement taxa; green shaded areas indicate the 0% propolis taxa and blue shaded indicate untreated control taxa; (**b**) Taxa value meeting a significant LDA threshold value of >4.0 are shown, which are displayed with a positive LDA score.

To identify the specific bacterial taxa associated with colitis alleviation by propolis treatment, we used a LEfSe analysis to compare gut microbiota (Figure 3). Total of 13 taxa showed significant differences in their relative abundance among untreated control, DSS (0% propolis) and 0.3% propolis groups (LDA threshold value log 10 > 4.0). Rats from the untreated control group showed higher abundance of bacterial class *Bacilli*, as well as its lower taxa Lactobacillales, Lactobacillaceae, and *Lactobacillus*. The DSS (0% propolis) group showed increases of potential pathogen bacteria abundance, like *Bacteroides* and Ruminococcaceae. At the phylum level, 0.3% propolis lead to increased Chloroflexi, Proteobacteria, Gemmatimonadetes. At the class level, 0.3% propolis supplement significantly increased the levels of Betaproteobacteria and Gemmatimonadetes. In addition, the relative abundance of order Gemmatimonadales, Xanthomonadales, as well as their lower taxa, *Gemmatimonadaceae and Xanthomonadaceae*, were significantly increased (Figure S1).

## 4. Discussion

IBD comprises a group of chronic intestinal inflammatory disorders that are now very common across the world. Current treatments for UC using anti-inflammatory or immunosuppressive drugs are often unable to sustain long-term clinical remission, which highlights the need for novel treatment therapies. The potential for diet to prevent or improve outcomes of the disease is still relatively poorly explored. Here, the ability of dietary intake of Chinese propolis in preventing DSS-induced colitis in rats was studied, including the role that modulation of the gut microbiota might play.

An increasing number of dietary components, such as bioactive dietary peptides, amino acids, prebiotic fibres, and polyunsaturated fatty acids, are recognized as having gut health benefits [18]. Many plant-derived phytochemicals, including polyphenolic compounds, seem especially beneficial and are often linked to anti-inflammatory and anti-oxidant activities. For instance, UC patients receiving a daily standardized anthocyanin-rich bilberry preparation for six weeks showed 63% remission [19]. A polyphenol-rich cranberry extract has also been found to help protect against diet-induced obesity, insulin resistance, and intestinal inflammation, which is associated with gut microbiota population changes [20]. Here, we used DSS to induce colitis in rodents, which is widely recognized as a model resembling human ulcerative colitis [12]. The group of untreated control animals was included in the first phase of the study solely as a reference point to demonstrate that treatment with DSS actually generated the symptoms of colitis. As anticipated, overt colitis was generated as shown by the DAI results and by the histology sections we included (as showed in Figure 1). We observed a lower weight gain and a higher colon length/weight value in the untreated control rats compared with the 0% propolis group (Supplementary Table S1). Interestingly, the present study showed that addition of 0.3% Chinese propolis extract to the diet was able to reduce the severity of DSS-induced colitis. This extract has been used previously in our studies and shown to have anti-inflammatory activities. Recently, we found that propolis may strengthen intestinal barrier function in Caco-2 cell monolayers by activating AMPK and ERK signalling. Moreover, rats fed with propolis (same batch and amount as used here) exhibited increased expression of the tight junction protein ZO-1 in the colonic epithelium [21]. Chinese propolis contains an abundance of polyphenolic compounds which may be responsible for these effects, including chrysin [22], kaempferol [23], apigenin [24], and caffeic acid [25], which have been demonstrated to prevent DSS-induced colitis. This also complements dietary supplementation studies carried out using Brazilian green propolis, which has been shown to alleviate colitis induced by acetic acid or trinitrobenzene sulfonic acid in rodents [26,27]. The Brazilian green propolis, with a local botanic origin of *Baccharis dracunculifolia* DC (Asteraceae), also has abundant phenolic acids, including caffeic acid and prenylated *p*-coumaric acids (Artepillin C and Baccarin). In contrast, the botanic origins of the Chinese propolis used in the present study is poplar trees (*Populus* sp.) and flavonoids and flavonoid esters are the main polyphenols in the propolis derived from this source [13]. Therefore, our findings presented here suggest there is a therapeutic potential of propolis for IBD irrespective of its geographical and botanical origins.

A disruption of gut microbiota populations is associated with many diseases, including IBD [28,29]. Such disruptions are also often linked to intestinal permeability changes which will facilitate the uptake of harmful agents [30]. In UC patients, microbial dysbiosis is generally characterized by reduced bacterial diversity and an increase in the ratio of Bacteroidetes/Firmicutes [31,32]. Shifts in microbiota are also observed in experimentally-induced colitis in animals. In our study, DSS-induced colitis was accompanied by shifts in gut microbiota populations, including the bacterial families Ruminococcaceae and Bacteroidaceae, which has also been found in previous studies [33]. In addition, when compared to normal (non-colitis) rats, there was a lower abundance of bacteria belonging to the order Lactobacillales, which comprises the lactic acid bacteria with well-known probiotic properties. A previous study which used the DSS-induced colitis rat model noted a similar change in lactic acid bacteria, finding that colonic injury was negatively correlated with Firmicutes, Actinobacteria, Lactobacillales, and *Lactobacillus* [2]. In addition, we also noticed that the SCFA values from the untreated control were lower than the DSS-challenged group, which is in contrast to a previously published result [34]. Nevertheless, some studies suggest that DSS challenge will boost the SCFA production [35,36]. 

Dietary propolis has shown potential to modulate gut microbial populations, but the evidence is limited. Propolis can inhibit the growth of some bacteria, especially pathogens, and has been examined for use in farm animals such as chickens and cows [37,38]. In a previous study in mice fed a high-fat diet, supplementation with Brazilian green propolis was found to have beneficial effects, stabilizing the gut microbiota profile. Longer-term treatment with propolis (0.2% in the diet) promoted increases in numbers of Bacteroides and *Helicobacter*, and reductions in numbers of *Oscillopira* and *Blautia* [39]. Our study, where rats were on a Western-style diet background, has now shown that supplementation with Chinese propolis (0.3%) leads to greater bacterial diversity and increases in Proteobacteria and Acidobacteria when compared to rats in the 0% propolis and non-DSS controls. Interestingly, increases in bacteria of the phyla Proteobacteria, such as *E. coli*, are regularly associated with IBD [40]. Given this, our observation of protection against colitis with a propolis treatment which also increases Proteobacteria seems counter-intuitive. One explanation is that some members of the phylum increased in response to propolis out-compete other more pathogenic members that could contribute to the inflammatory processes of the disease. Acidobacteria are widely detected by 16S rRNA sequencing in different environments; nevertheless, their roles or physiological activities during colitis remain elusive [41]. Our finding that propolis supplementation during DSS-induced colitis increases Acidobacteria, suggesting that Acidobacteria might have an important and beneficial role in gut health which should be explored further. Using LEfSe analysis to examine differences in the relative abundances of microbial taxa, the 0.3% propolis treatment resulted in a trend of a return to non-DSS treatment abundances for the Bacteroides and Bacteroidaceae. The lowering of the abundances of these microbes could also potentially explain the reduced colitis severity seen with the 0.3% propolis as these bacteria have been linked to poor gut health outcomes and often highly abundant in colitis patients [42]. 

## 5. Conclusions

In conclusion, our studies show for the first time that dietary supplementation with Chinese propolis can protect against DSS-induced colitis in rats consuming a Western-style diet. Increases in gut microbiota, including bacteria of the phyla Proteobacteria and Acidobacteria, may contribute to the protection. Clinical investigations are warranted to assess whether our promising findings can be translated into a therapy for IBD.

## Figures and Tables

**Table 1:** Composition of control (Western) diet.

Components	g/kg Diet
Casein	250
Cornstarch	350
Sucrose	100
Fat Blend (Canola and Palm oils)	200
Wheat bran	50
l-Cystine	3
Choline bitartrate	2.5
Vitamins (AIN 93)	10
Minerals	35
Tert-butyl hydroquinol	0.014

**Table 2:** Organ and fat pad weights ^1^.

Indices	0% Propolis	0.1% Propolis	0.2% Propolis	0.3% Propolis
Total fat pad (mg/g)	20.2 ± 1.3	18.4 ± 1.0	23.0 ± 2.4	20.4 ± 1.1
Liver (mg/g)	40.8 ± 1.3	38.5 ± 0.8	43.1 ± 2.1	40.8 ± 1.1
Spleen(mg/g)	2.7 ± 0.1	2.6 ± 0.1	2.9 ± 0.2	2.5 ± 0.1
Kidney (mg/g)	7.6 ± 0.2	7.4 ± 0.2	7.8 ± 0.4	7.4 ± 0.2

^1^ Data are expressed as means ± SEM (*n* = 8). Fat pad weights were measured as the sum of the epididymal, retroperitoneal, and mesenteric fat pads.

**Table 3:** Effects of propolis administration on individual and total SCFA levels in caecal digesta of DSS-induced colitis rats ^1^.

Variables	Treatment			
Cecum weights, g	0% propolis	0.1% propolis	0.2% propolis	0.3% propolis
Tissue	0.7 ± 0.0	0.7 ± 0.0	0.7 ± 0.0	0.7 ± 0.0
Digesta	1.8 ± 0.2	1.7 ± 0.1	1.8 ± 0.2	1.8 ± 0.1
Cecum pH	7.7 ± 0.1	7.7 ± 0.1	7.9 ± 0.1	7.7 ± 0.1
Pool, μmol	0% propolis	0.1% propolis	0.2% propolis	0.3% propolis
Acetate	79.6 ± 6.9 ^a,b^	89.5 ± 5.3 ^a^	66.8 ± 4.1 ^b^	74.9 ± 3.5 ^a,b^
Propionate	20.3 ± 1.4	19.5 ± 0.8	17.2 ± 0.8	19.2 ± 0.9
Butyrate	13.0 ± 1.1	12.6 ± 1.3	12.1 ± 0.9	12.7 ± 0.8
Total SCFA	116.1 ± 8.9 ^a,b^	124.7 ± 4.2 ^a^	99.3 ± 5.1 ^b^	110.1 ± 4.7 ^a,b^
Percentage of total, %	0% propolis	0.1% propolis	0.2% propolis	0.3% propolis
Acetate	68.2 ± 1.1	71.4 ± 2.1	67.0 ± 1.5	68.1 ± 0.8
Propionate	17.7 ± 0.7	15.8 ± 0.9	17.5 ± 0.8	17.5 ± 0.5
Butyrate	11.3 ± 0.7	10.3 ± 1.2	12.2 ± 0.8	11.5 ± 0.4

^1^ Data are expressed as means ± SEM (*n* = 8). The means with different letters are significantly different means without a common letter differ, *p* < 0.05.

## Supplementary Materials

The following are available online at www.mdpi.com/2072-6643/9/8/875/s1, Figure S1 LEfSe comparison of the relative abundances of gut microbiota in the 0% propolis, 0.3% propolis, and untreated control groups; Table S1 Weight gain, colon length/weight, organ and fat pad weights of untreated control rats; Table S2 Individual and total SCFA levels in normal rats.
