# Supplementary material for: Dietary Propolis Ameliorates Dextran Sulfate Sodium-Induced Colitis and Modulates the Gut Microbiota in Rats Fed a Western Diet
Source: Nutrients. 2017 Aug 14;9(8):875. doi: 10.3390/nu9080875 (PMC5579668; doi:10.3390/nu9080875)
Supplement: Supplementary file 1 [file nutrients-09-00875-s001.zip › nutrients-214320-supplementary-r2.pdf]

Figure S1. LEfSe comparison of the relative abundances of gut microbiota in the 0% propolis, 0.3% propolis and untreated control groups. The taxonomic cladogram was derived from LEfSe analysis of 16S sequences. Class CP means 0.3% propolis, Class DSS means 0% propolis and Class N means untreated control group. Only taxa meeting an LDA score threshold > 4.0 are listed and their relative abundances are shown. Relative abundance of a bacterial type is shown as the percentage of total OTU counts. The straight line shows the group average abundance and the dashed line shows the group median abundance.

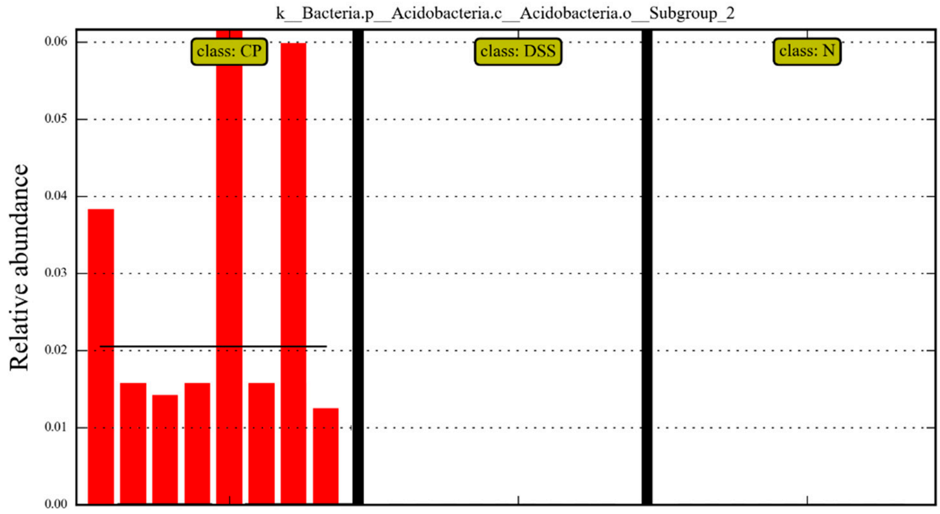

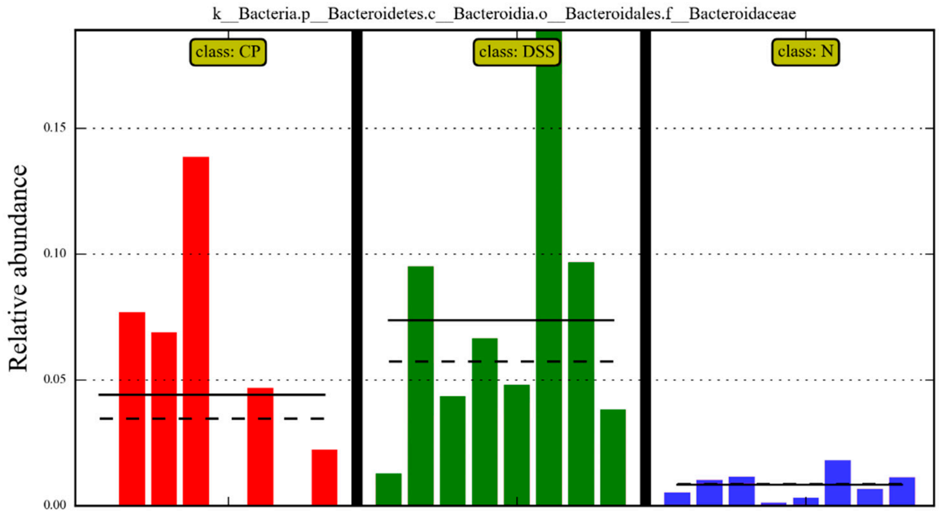

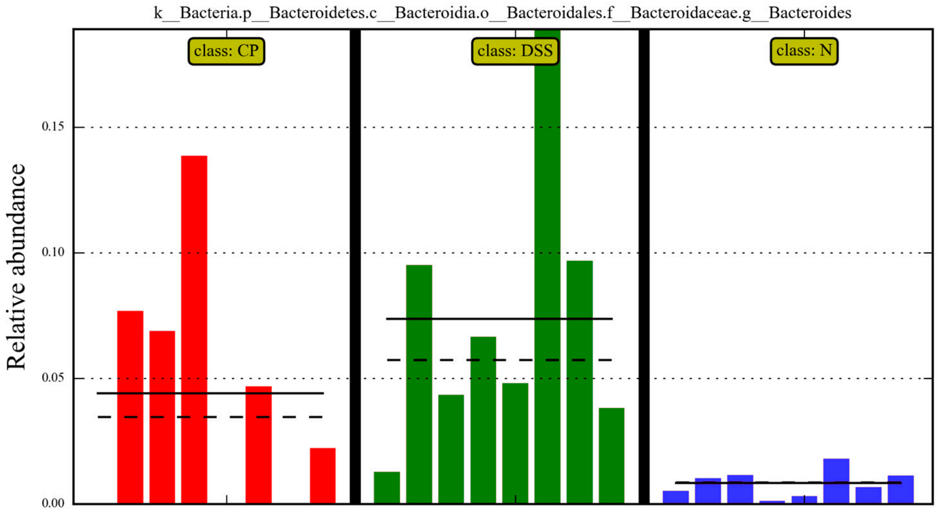
k__Bacteria.p__Bacteroidetes.c__Bacteroidia.o__Bacteroidales.f__Bacteroidaceae.g__Bacteroides
class: CP
class: DSS
class: N
Relative abundance
0.15
0.10
0.05
0.00

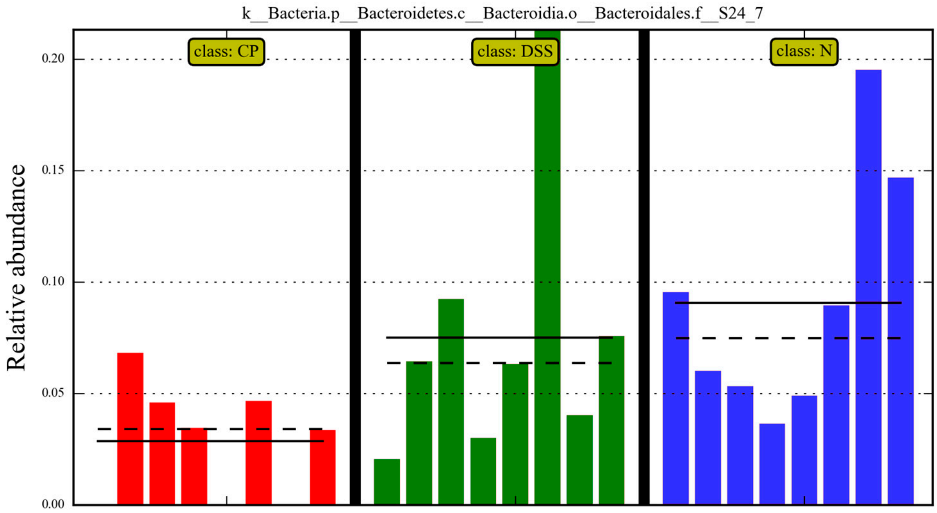
k__Bacteria.p__Bacteroidetes.c__Bacteroidia.o__Bacteroidales.f__S24_7
class: CP
class: DSS
class: N
Relative abundance
0.20
0.15
0.10
0.05
0.00

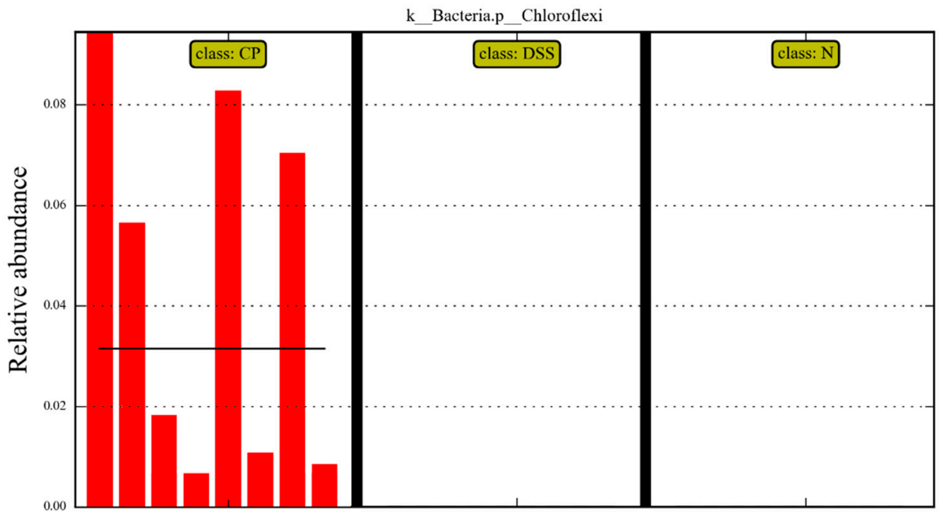
k__Bacteria.p__Chloroflexi
class: CP
class: DSS
class: N
Relative abundance
0.08
0.06
0.04
0.02
0.00

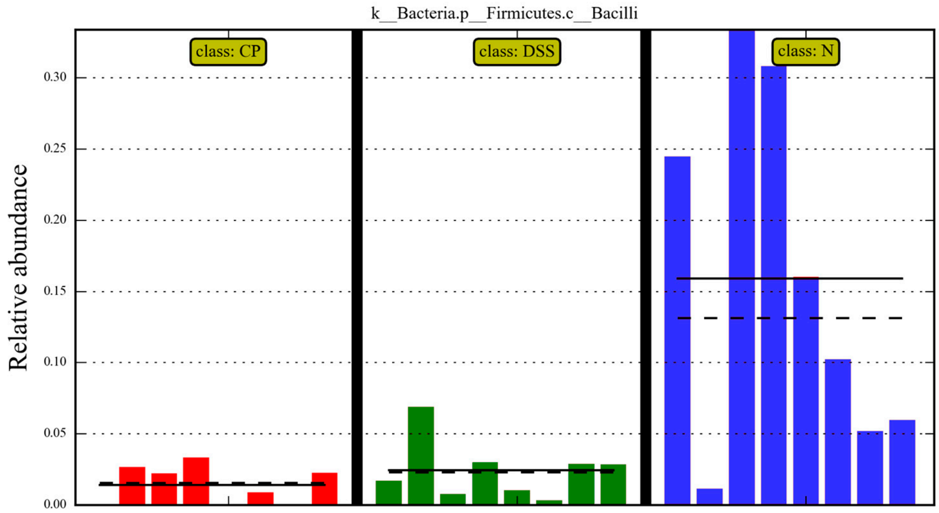
k__Bacteria.p__Firmicutes.c__Bacilli
class: CP
class: DSS
class: N
Relative abundance
0.30
0.25
0.20
0.15
0.10
0.05
0.00

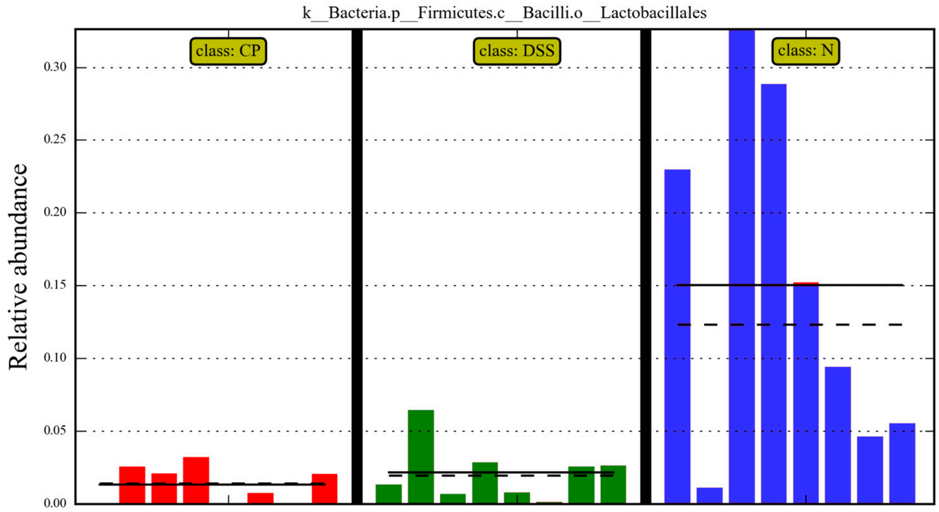
k__Bacteria.p__Firmicutes.c__Bacilli.o__Lactobacillales
Relative abundance
0.00
0.05
0.10
0.15
0.20
0.25
0.30
class: CP
class: DSS
class: N

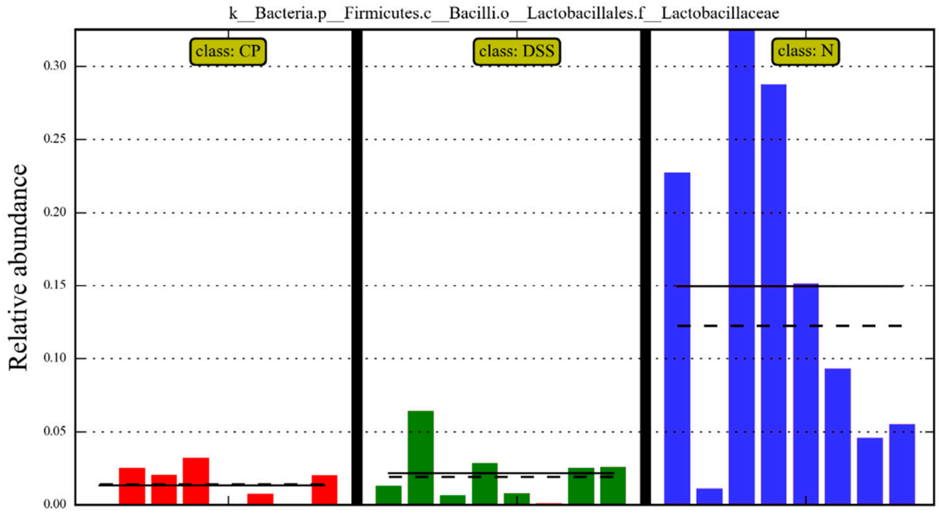
k__Bacteria.p__Firmicutes.c__Bacilli.o__Lactobacillales.f__Lactobacillaceae
Relative abundance
0.00
0.05
0.10
0.15
0.20
0.25
0.30
class: CP
class: DSS
class: N

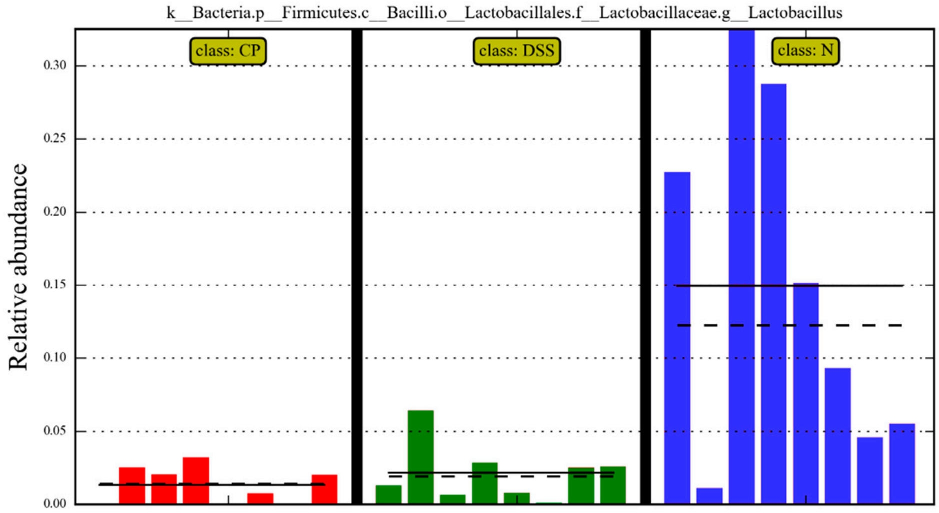
k__Bacteria.p__Firmicutes.c__Bacilli.o__Lactobacillales.f__Lactobacillaceae.g__Lactobacillus
class: CP
class: DSS
class: N
Relative abundance
0.30
0.25
0.20
0.15
0.10
0.05
0.00

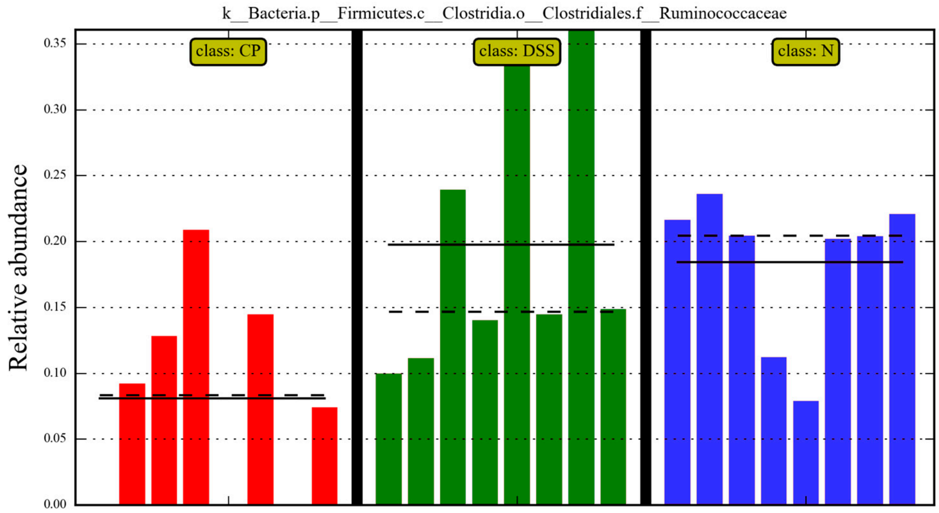
k__Bacteria.p__Firmicutes.c__Clostridia.o__Clostridiales.f__Ruminococcaceae
class: CP
class: DSS
class: N
Relative abundance
0.35
0.30
0.25
0.20
0.15
0.10
0.05
0.00

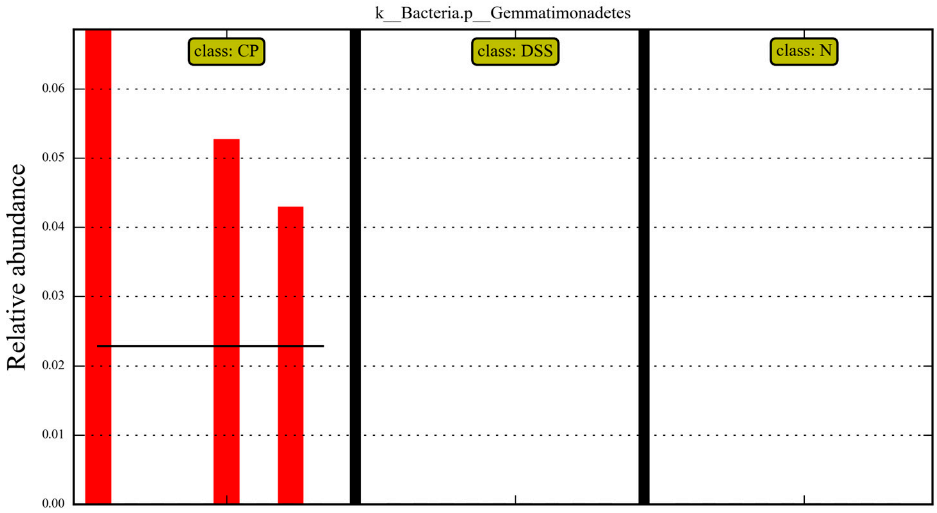
k__Bacteria.p__Gemmatimonadetes
class: CP
class: DSS
class: N
Relative abundance
0.00
0.01
0.02
0.03
0.04
0.05
0.06

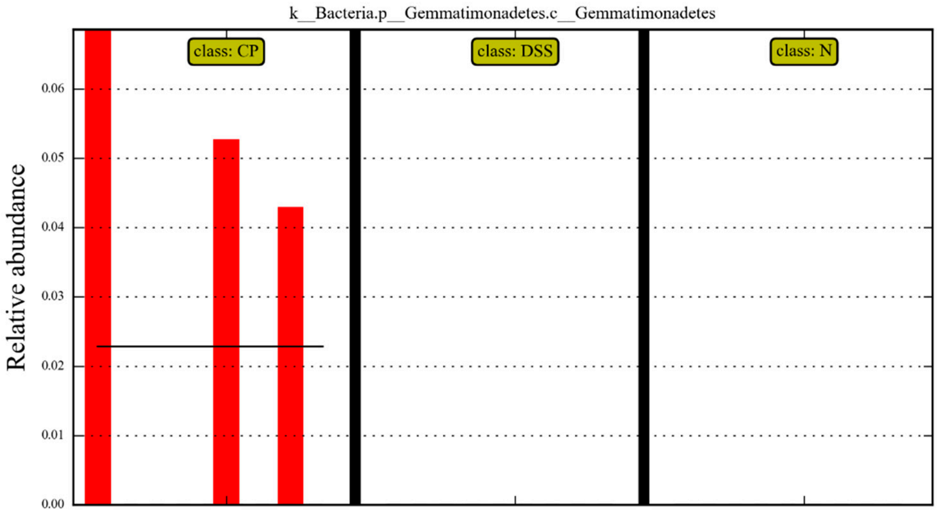
k__Bacteria.p__Gemmatimonadetes.c__Gemmatimonadetes
class: CP
class: DSS
class: N
Relative abundance
0.00
0.01
0.02
0.03
0.04
0.05
0.06

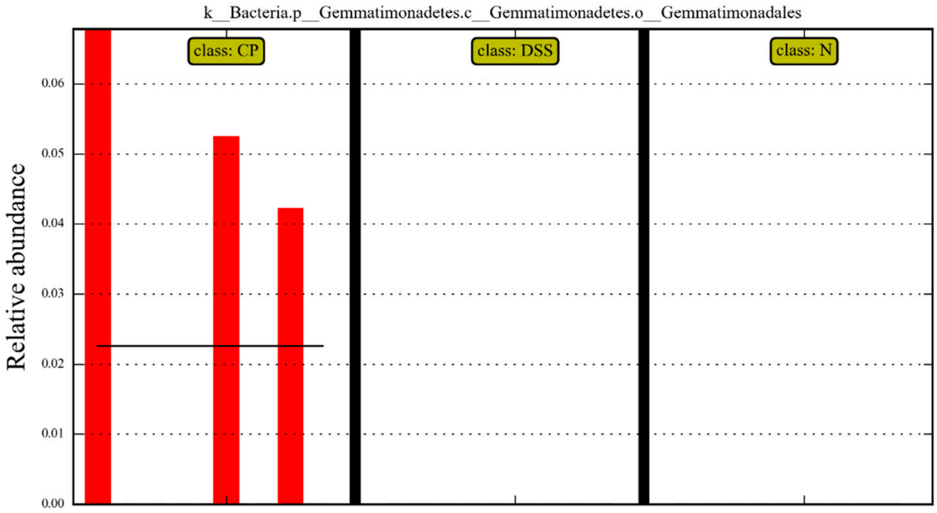
k__Bacteria.p__Gemmatimonadetes.c__Gemmatimonadetes.o__Gemmatimonadales
class: CP
class: DSS
class: N
Relative abundance
0.06
0.05
0.04
0.03
0.02
0.01
0.00

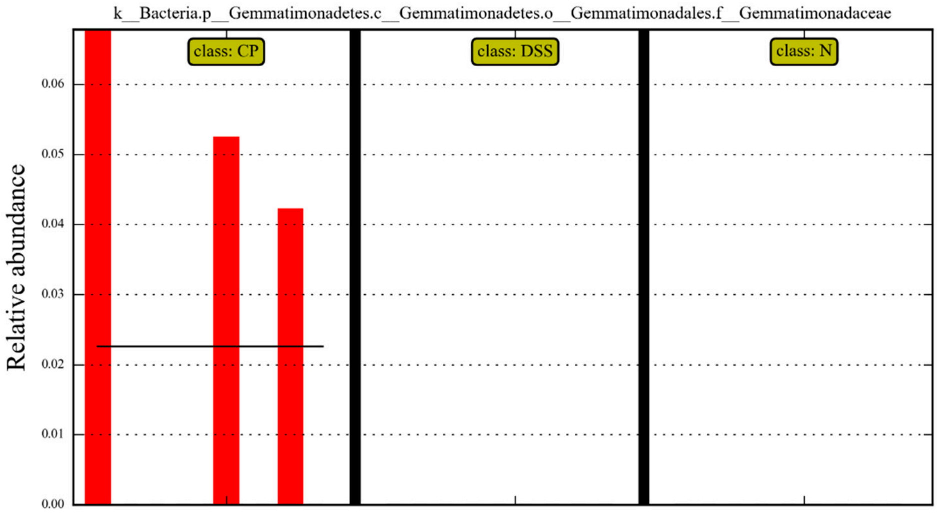
k__Bacteria.p__Gemmatimonadetes.c__Gemmatimonadetes.o__Gemmatimonadales.f__Gemmatimonadaceae
class: CP
class: DSS
class: N
Relative abundance
0.06
0.05
0.04
0.03
0.02
0.01
0.00

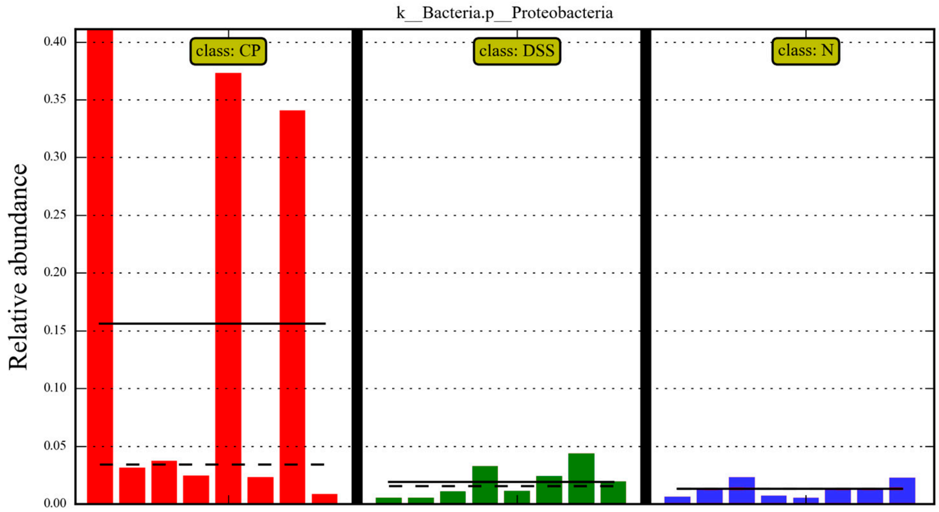
k__Bacteria.p__Proteobacteria
class: CP
class: DSS
class: N
Relative abundance
0.40
0.35
0.30
0.25
0.20
0.15
0.10
0.05
0.00

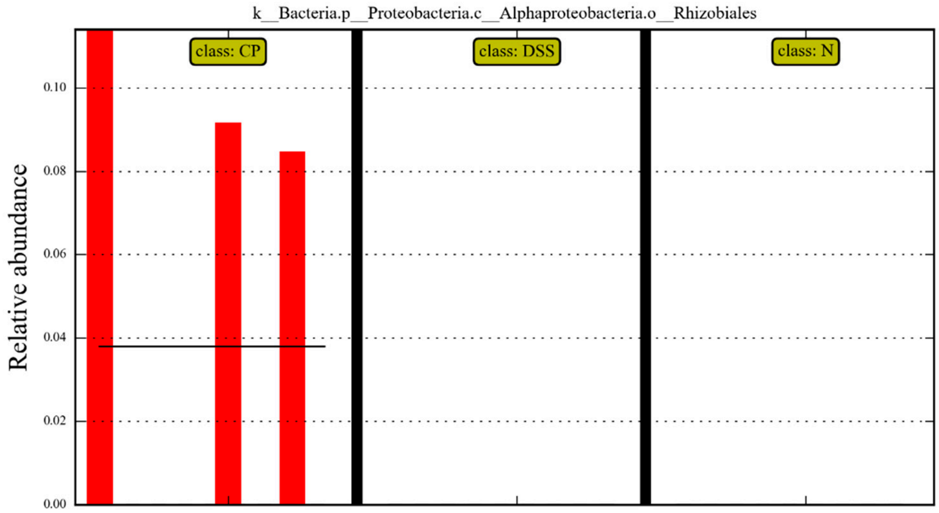
k__Bacteria.p__Proteobacteria.c__Alphaproteobacteria.o__Rhizobiales
class: CP
class: DSS
class: N
Relative abundance
0.10
0.08
0.06
0.04
0.02
0.00

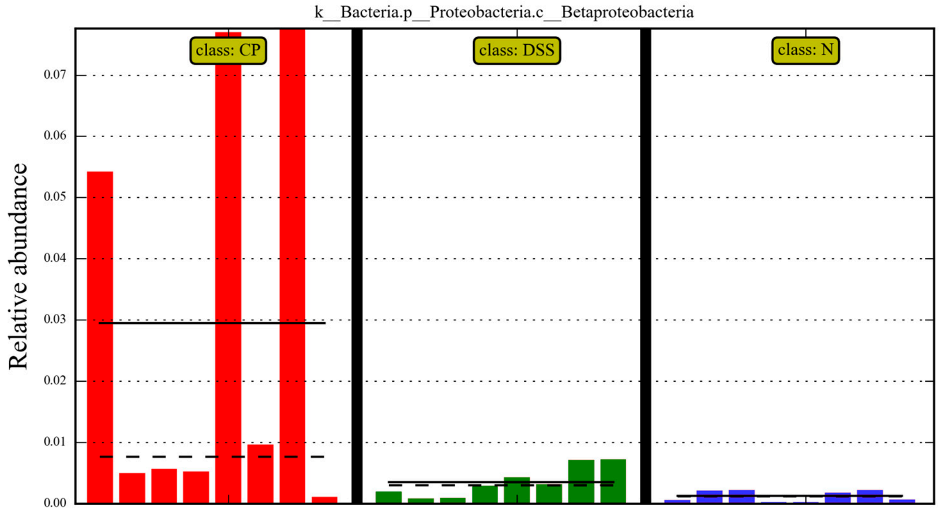
k__Bacteria.p__Proteobacteria.c__Betaproteobacteria
class: CP
class: DSS
class: N
Relative abundance
0.00
0.01
0.02
0.03
0.04
0.05
0.06
0.07

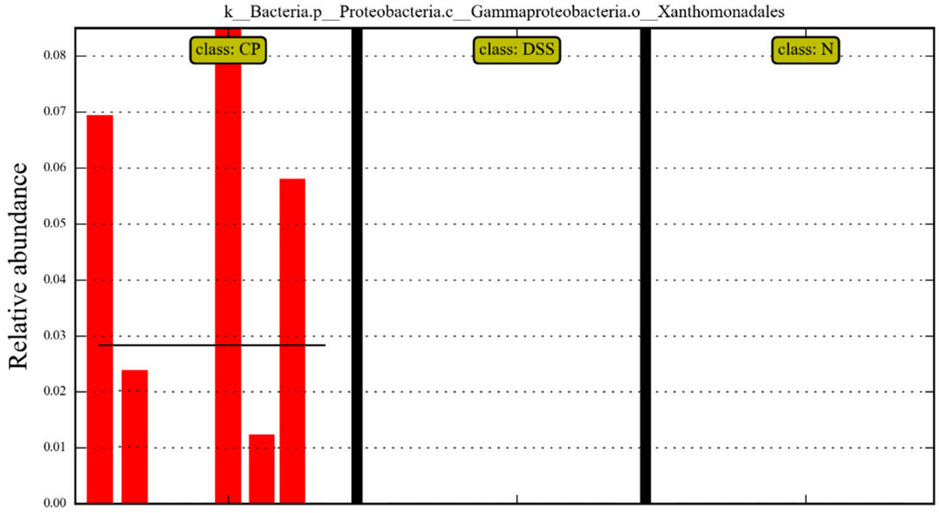
k__Bacteria.p__Proteobacteria.c__Gammaproteobacteria.o__Xanthomonadales
class: CP
class: DSS
class: N
Relative abundance
0.00
0.01
0.02
0.03
0.04
0.05
0.06
0.07
0.08

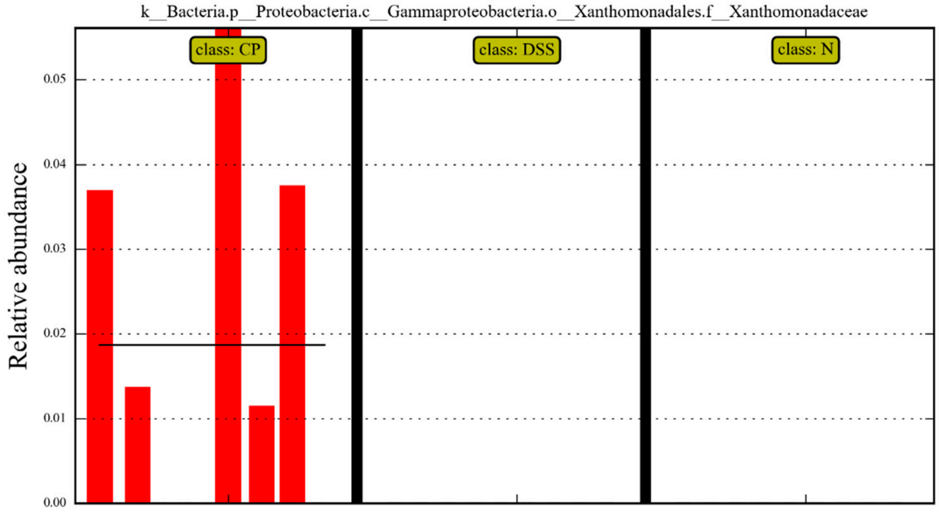
k__Bacteria.p__Proteobacteria.c__Gammaproteobacteria.o__Xanthomonadales.f__Xanthomonadaceae
class: CP
class: DSS
class: N
Relative abundance
0.00
0.01
0.02
0.03
0.04
0.05

Supplementary Table S1. Weight gain, colon length/weight, organ and fat pad weights of untreated control rats [1].

Indices	Untreated Control
Weight gain (g)	72.6 ± 6.7
Colon length/weight (mm/g)	52.42 ± 1.5
Total fat pad (mg/g)	27.2 ± 0.6
Liver (mg/g)	35.5 ± 0.3
Spleen(mg/g)	2.3 ± 0.0
Kidney (mg/g)	7.1 ± 0.2

[1] Data are expressed as means ± SEM (n = 5). Fat pad weights were measured as the sum of the epididymal, retroperitoneal and mesenteric fat pads.

Supplementary Table S2. Individual and total SCFA levels in normal rats[1].

Variables	Treatment
Cecum weights, g	Untreated Control
Tissue	0.7 ± 0.1
Digesta	1.8 ± 0.1
Cecum pH	7.8 ± 0.1
Pool, μmol	Untreated Control
Acetate	60.8 ± 9.4
Propionate	17.4 ± 1.6
Butyrate	13.4 ± 2.6
Total SCFA	96.7 ± 11.9
Percentage of total, %	Untreated Control
Acetate	62.3 ± 1.5
Propionate	18.1 ± 0.6
Butyrate	13.9 ± 1.1

[1] Data are expressed as means ± SEM (n = 5).
